# Combined Analysis of the Metabolome and Transcriptome to Explore Heat Stress Responses and Adaptation Mechanisms in Celery (*Apium graveolens* L.)

**DOI:** 10.3390/ijms23063367

**Published:** 2022-03-20

**Authors:** Mengyao Li, Jie Li, Ran Zhang, Yuanxiu Lin, Aisheng Xiong, Guofei Tan, Ya Luo, Yong Zhang, Qing Chen, Yan Wang, Yunting Zhang, Xiaorong Wang, Haoru Tang

**Affiliations:** 1 College of Horticulture, Sichuan Agricultural University, Chengdu 611130, China; limy@sicau.edu.cn (M.L.); lijie@stu.sicau.edu.cn (J.L.); zr_807@163.com (R.Z.); linyx@sicau.edu.cn (Y.L.); 13621@sicau.edu.cn (Y.L.); zhyong@sicau.edu.cn (Y.Z.); supnovel@sicau.edu.cn (Q.C.); wangyanwxy@sicau.edu.cn (Y.W.); asyunting@sicau.edu.cn (Y.Z.); wangxr@sicau.edu.cn (X.W.); 2 Institute of Pomology & Olericulture, Sichuan Agricultural University, Chengdu 611130, China; 3 College of Horticulture, Nanjing Agricultural University, Nanjing 210095, China; xiongaisheng@njau.edu.cn; 4 Institute of Horticulture, Guizhou Academy of Agricultural Sciences, Guiyang 550006, China; tgfei@foxmail.com

**Keywords:** celery, heat stress, leaf morphological traits, physiological mechanisms, metabolic pathways, gene expression level, genetic associations

## Abstract

Celery is an important leafy vegetable that can grow during the cool season and does not tolerate high temperatures. Heat stress is widely acknowledged as one of the main abiotic stresses affecting the growth and yield of celery. The morphological and physiological indices of celery were investigated in the present study to explore the physiological mechanisms in response to high temperatures. Results showed that the antioxidant enzyme activity, proline, relative conductivity, and malondialdehyde were increased, while chlorophyll and the water content of leaves decreased under high-temperature conditions. Short-term heat treatment increased the stomatal conductance to cool off the leaves by transpiration; however, long-term heat treatment led to stomatal closure to prevent leaf dehydration. In addition, high temperature caused a disordered arrangement of palisade tissue and a loose arrangement of spongy tissue in celery leaves. Combined metabolomic and transcriptomic analyses were further used to reveal the regulatory mechanisms in response to heat stress at the molecular level in celery. A total of 1003 differential metabolites were identified and significantly enriched in amino acid metabolism and the tricarboxilic acid (TCA) cycle. Transcriptome sequencing detected 24,264 different genes, including multiple transcription factor families such as HSF, WRKY, MYB, AP2, bZIP, and bHLH family members that were significantly upregulated in response to heat stress, suggesting that these genes were involved in the response to heat stress. In addition, transcriptional and metabolic pathway analyses showed that heat stress inhibited the glycolysis pathway and delayed the TCA cycle but increased the expression of most amino acid synthesis pathways such as proline, arginine, and serine, consistent with the results of physiological indicators. qRT-PCR further showed that the expression pattern was similar to the expression abundance in the transcriptome. The important metabolites and genes in celery that significantly contributed to the response to high temperatures were identified in the present study, which provided the theoretical basis for breeding heat-resistant celery.

## 1. Introduction

Temperature is an important environmental factor that affects plant growth and yield, and its effect has been emphasized with global warming. Studies have shown that heat stress can disrupt the homeostasis of plant cells and damage chlorophyll and the photosystem, causing substantial plant damage, insufficient energy supply, and stunted plant growth [1]. It is widely acknowledged that plant response to heat stress involves complex molecular and biochemical mechanisms. When plants sense high temperatures, they can induce antioxidant enzymes such as superoxide dismutase (SOD), peroxidase (POD), and catalase (CAT) to produce antioxidants to remove excess reactive oxygen species (ROS) in the body [2]. In addition, the synthesis and decomposition pathways of various compounds such as amino acids, proteins, sugars, and lipids are altered, leading to the accumulation of secondary metabolites [3,4]. These compounds act as antioxidants, osmoprotectants, intermediates, and stress and signaling molecules under heat stress to maintain the normal life activities of plants in response to adverse stress [5].

The molecular mechanisms of heat stress responses in plants are complex and depend on diverse signal transduction pathways, metabolites, and genes [6,7]. In recent years, a series of high temperature-related genes, small RNAs, and proteins have been reported. Importantly, it has been shown that high temperatures can affect celery photosynthesis and downregulate genes related to chlorophyll synthesis, such as *AgHEMA* and *AgCHLH* [8]. Moreover, the regulation network of *miR156*/*SPL13* can improve the heat tolerance of alfalfa [9]. Overexpression *MdATG18a* in apple improves basal thermotolerance, increases antioxidant activity and minimizes damage to the photosynthetic system [10]. Various plants respond to heat stress by activating the heat shock response with the help of molecular chaperones (heat shock proteins, HSPs), which can help to prevent protein misfolding and aggregation at high temperatures and stabilize intracellular proteins and cellular homeostasis [11]. Multiple studies have reported the critical role of HSPs in sensing and transmitting heat stress signals [12]. These HSPs are regulated by activating heat shock transcription factors (HSFs) to enhance heat tolerance in plants [13]. Knockout of *HsfA1* in Arabidopsis and tomato yielded heat stress-sensitive phenotypes and decreased the expression of many heat shock-responsive genes [14]. In addition, studies found that various transcription factors such as bZIP, DREB, MYB and WRKY also play a role in the heat response mechanism [15,16,17]. These studies suggest that the regulatory mechanisms of plant tolerance to heat stress may involve multi-signaling and multi-regulatory genes.

Exploring the molecular mechanisms of plant response to heat stress can improve the current understanding of genetic plant thermotolerance. Although several regulatory pathways have been documented in heat stress response, there is little doubt that more regulatory pathways have been unexplored. A deeper understanding of the stress response system could be obtained by integrating different approaches such as physiological, metabolomic, and transcriptomic data [18]. Transcriptome and metabolomic analysis can link the genotype and phenotype of plants, providing crucial information for the study of metabolites and regulatory pathways related to plant adaptation to high-temperature stress. Comparative transcriptome combined with metabolome analyses revealed that the thermotolerance of tall fescue might be related to the expression of heat-responsive genes enriched in fatty acid and sugar metabolism pathways [19]. Metabolomic and transcriptomic sequencing have shown that the glutathione metabolic pathway plays a key role in the heat stress response of peppers [20]. Glaubitz et al. [21] revealed that high nighttime temperatures could activate the tricarboxilic acid (TCA) cycle, with increased levels in pathways branching off. The combined analysis of the transcriptome and metabolome can provide comprehensive information on the networks involved in regulating heat stress responses and provide a powerful analytical method for elucidating the changes from the genotype to the phenotype in plants under heat stress.

Celery (*Apium graveolens* L.) is an important biennial leaf vegetable crop of the Apiaceae family, which is rich in nutritional and medicinal compounds and is widely cultivated throughout the world [22]. However, it has been established that celery prefers cold environments to hot surroundings. The optimal temperature for growth is 15–20 °C, and growth and development are affected at temperatures above 26 °C [23]. High temperatures can lead to physiological and biochemical disorders, inducing severe leaf wilting or chlorosis and reducing yield and quality. Global warming contributes to higher temperatures, especially in summer, significantly restricting celery growth and production in summer-autumn seasons. Although heat stress-related genes in celery have been identified [24,25], there is no systematic study on the mechanisms underlying celery response to heat stress. To increase our understanding of response mechanisms to heat stress in celery, a study on potted celery plants was conducted in growing chambers under heat stress that lasted up to 24 h. Changes at the molecular level were investigated and linked to the adaptive responses of celery including physiological, anatomical, metabolic, and transcriptional traits. Understanding the physiological characteristics and identifying key genes and metabolites in celery associated with high-temperature stress is vital for breeding new heat-resistant cultivars, especially with global climate change.

## 2. Results

### 2.1. Analysis of Physiological Characteristics of Celery under Heat Stress

**Figure 1:**
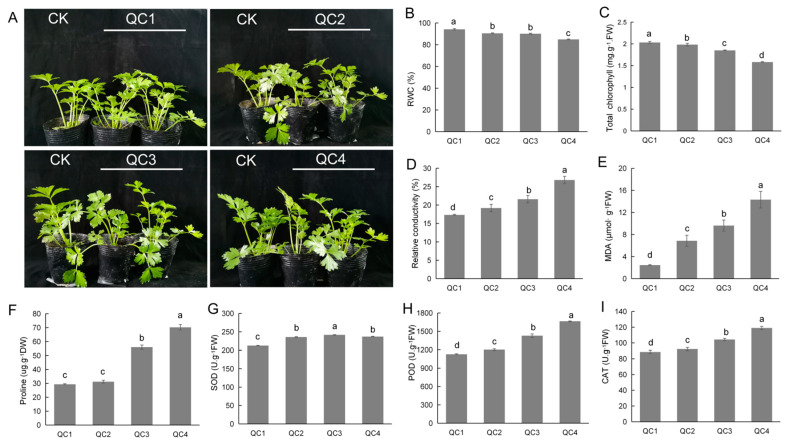
Effect of heat stress on celery growth. (**A**) Phenotypic traits of celery in different time periods under heat stress. (**B**–**I**) The effects of heat stress on physiological traits of celery. Relative water content (RWC) (**B**), chlorophyll content (**C**), relative electrical conductivity (**D**), malondialdehyde (MDA) (**E**), proline (**F**), superoxide dismutase (SOD) (**G**), peroxidase (POD) (**H**), and catalase (CAT) (**I**) of celery under heat stress. Each bar represents the mean ± SD, *T*-test at the significance level of 0.05 (*p* < 0.05), different letters (a, b, c) indicate significant difference, and the same letters indicate insignificant differences.

Celery exhibited different degrees of tolerance and sensitivity for different periods of heat stress. Figure 1A shows the growth of celery at 0 h (QC1), 1 h (QC2), 6 h (QC3), and 24 h (QC4) at a high temperature of 38 °C. During analysis of the celery phenotype, the leaves of celery seedlings in the QC2 and QC3 stages showed wilting and lodging, while wilting was relatively decreased in the QC4 stage.

The relative water content and chlorophyll concentration were significantly reduced (Figure 1B,C). In contrast, the relative electrical conductivity increased gradually after heat treatment (Figure 1D). The content of malondialdehyde (MDA) induced by lipid peroxidation of polyunsaturated fatty acids and the cell osmotic regulator proline were significantly increased (Figure 1E,F). The main reactive oxygen species (ROS) scavenging enzymes, SOD, POD and CAT, were significantly increased after heat treatment, and SOD activity exhibited an increasing and decreasing trend (Figure 1G–I). These results indicate that celery undergoes physiological changes in response to heat stress and activates the defense system to protect internal tissue.

### 2.2. Changes in Stomatal Movement and Cross-Section of Celery Leaves under Heat Stress

**Figure 2:**
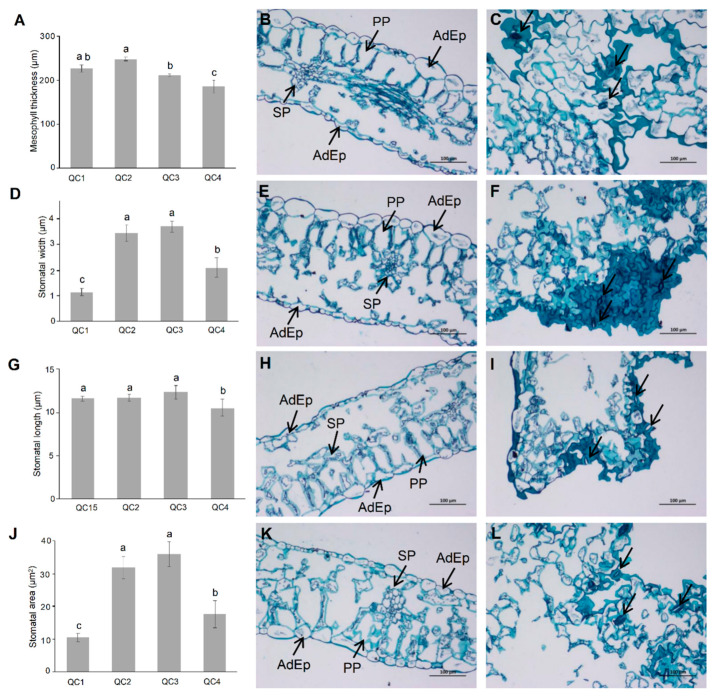
The mesophyll thickness (**A**), stomatal length (**D**), stomatal width (**G**), stomatal area (**J**), cross section plot (**B**,**E**,**H**,**K**), and flat section plot (**C**,**F**,**I**,**L**) of celery at four different stages under heat stress. The arrows of the figures (**C**,**F**,**I**,**L**) point to the stomata. SP: sponge tissue; PP: palisade tissue; AdEp: epidermal cell layer. Each bar represents the mean ± SD, *T*-test at the significance level of 0.05 (*p* < 0.05), different letters (a, b, c) indicate significant difference, and the same letters indicate insignificant differences.

The anatomical characteristics were compared under different heat stress treatments (Figure 2). As shown in Figure 2B,E,H,K, the variation of mesophyll thickness for 1 h after heat treatment (QC2) was not significant, but gradually decreased significantly in QC3 and QC4 stages, and even decreased significantly in QC3 and QC4 stages to be lower than QC1 (Figure 2A). Compared with QC1, the palisade cells in QC2 are disordered and the spongy tissue is loose. In addition, in QC3 and QC4 stages, the spongy tissues were closely arranged, indicating that long-term heat stress leads to thinning of mesophyll thickness. The stomatal width and stomatal area (Figure 2C,F,I,L) increased in QC2 and QC3 stages. The stomatal width and stomatal area of QC4 were lower than those of QC2 and QC3, but higher than those of QC1 (Figure 2D,J). The stomatal length (Figure 2G) did not change much after heat treatment for 1 h (QC2) and 6 h (QC3), but decreased significantly after heat treatment for 24 h (QC4). One hour of heat treatment (QC2) led to slight stomata opening that further increased after 6 h of treatment (QC3); such stomatal opening occurring with a reduced level of relative water content remained similar in QC2 and QC3. After 24 h of heat treatment (QC4), stomata opening was reduced but remained larger than without heat treatment (QC1) leading to severe water loss in leaves and wilting (Figure 1A,B).

### 2.3. Metabolomic Analysis of Celery in Response to Heat Stress

#### 2.3.1. Overall Analysis of Metabolite Components

**Figure 3:**
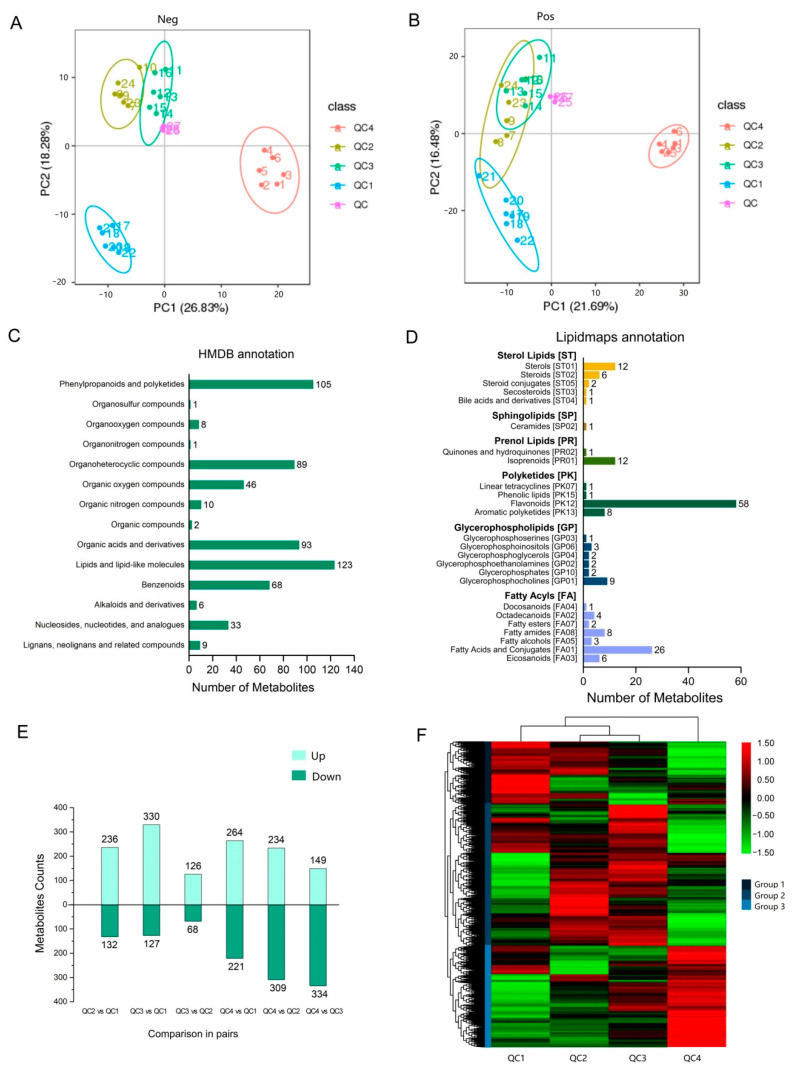
Identification of accumulated metabolites under heat treatment in celery. (**A**) Principal component analysis (PCA) score plots among four celery samples and QC sample in negative ion modes. (**B**) PCA score plots among four celery samples and QC sample in positive ion modes. (**C**) The metabolites classified through the HMDB database. (**D**) The metabolites classified through the Lipid Maps database. (**E**) Comparison of upregulated and downregulated metabolites under different heat treatments. (**F**) Hierarchical clustering analysis of differential metabolite expression for all samples, and the logarithmic quantitative results of metabolites are row scale.

UHPLC-MS/MS (ultra-high-performance liquid chromatography-tandem mass spectrometry hyphenated with high resolution mass spectrometer) was used to investigate the metabolite changes in celery that develop resistance to heat. Principal component analysis (PCA) was used to analyze the data in positive and negative ion modes (Figure 3A,B). We plotted the 27 samples (including 3 quality control samples and 24 test samples) in PCA diagrams for the positive and negative ion modes using different colors according to their heat treatment. PCA multi-space of axis 1 and 2 for negative ion samples explained ~45% of the total variation and assist to separate the treatments without any overlap (Figure 3A). Samples of the positive ion were mostly separated, however, samples of QC2 and QC3 showed almost complete overlap suggesting high similarity. In addition, the quality control (QC) samples on the PCA analysis diagram were clustered in four groups that were clearly separated, indicating the high dissimilarity of celery among heat treatment, where QC1 and QC4 samples occupied the extreme ends of PC1 while only QC2 and QC3 samples were in the positive end of PC2. The metabolite composition of QC2 and QC3 was similar and even showed a substantial overlap in the positive ion.

A total of 1472 metabolites were detected in celery samples at four high-temperature stages, and the identified metabolites were annotated using the HMDB and Lipidmaps databases. A total of 594 metabolites were classified into 14 categories in the HMDB database (Figure 3C), the most enriched types were lipids and lipid-like molecules (123 metabolites), followed by phenylpropanoids and polyketides (105 metabolites), organic acids and derivatives (93 metabolites), and organoheterocycle compounds (89 metabolites). In the Lipidmaps database (Figure 3D), the most diverse types were fatty acyls (7 categories and 50 metabolites), followed by glycerophospholipids (6 categories and 19 metabolites), and polyketides in the database consisting of the most metabolites (4 categories and 68 metabolites).

#### 2.3.2. Differential Metabolite Analysis

A total of 1003 metabolites were differentially expressed during pairwise comparison (Figure 3E). The most divergent were QC4 vs. QC2, which produced the highest number of differential metabolites (*n* = 543; 243 up-regulated and 309 down-regulated), followed by QC4 vs. QC1 (*n* = 485) and QC4 vs. QC3 (*n* = 483), where the most similar groups were QC2 and QC3, which had the least number of differential metabolites (*n* = 194). The metabolite abundance was normalized, then hierarchical clustering analysis was performed on all differential metabolites. As shown in Figure 3F, the cluster analysis of metabolites from four treatments exhibited a clear grouping pattern; all differential metabolites were divided into three groups. Most metabolites were highly expressed in QC3, followed by QC2. The clustering heatmap showed that QC2 and QC3 were clustered, indicating similarity between them. QC2 showed high expression of most group 2 metabolites and over 1/2 of group 1 metabolites, and moderate expression of a small fraction of group 3. QC3 showed a similar trend to QC2, but in moderate expression in group 1 and increased metabolites with high expression in group 3. QC1 plants showed high expression of most group 1 metabolites and over 1/3 of group 2 metabolites and moderate expressed some metabolites of group 3. On the opposite, QC4 plants highly expressed all group 3 metabolites and only moderately expressed a few metabolites from groups 1 and 2. It shows that the metabolite expression levels of QC2 and QC3 are similar, while the metabolite expression levels of QC1 and QC4 have the largest difference.

### 2.4. Transcriptomic Analysis of Celery in Response to Heat Stress

#### 2.4.1. Transcriptome Sequencing and Whole Gene Expression Analysis

**Figure 4:**
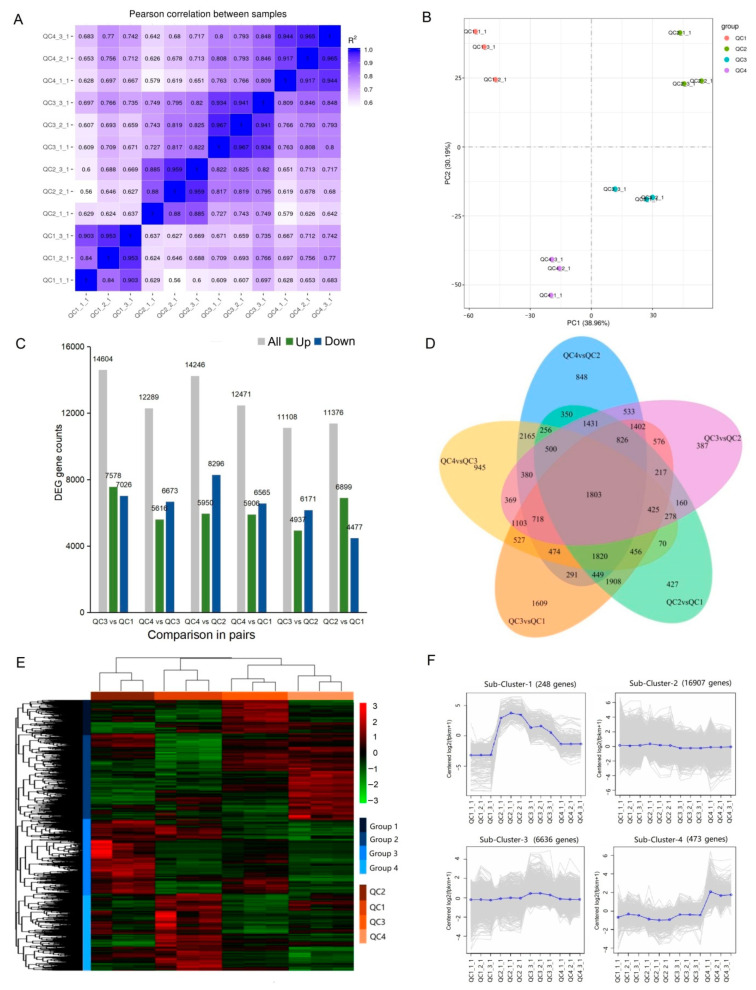
Analysis of gene expression under heat treatment in celery. (**A**) Pearson correlation coefficients (PCCs) of gene expression under different heat treatments (QC1, QC2, QC3 and QC4) for three replicates presented with _1, _2 and _3. (**B**) PCA score plots of transcriptome data for four celery samples. (**C**) Number of DEGs at any two different treatments. The numbers of up- and down-regulated genes are represented by green and blue bars, respectively. (**D**) Venn diagram of DEGs commonly and uniquely expressed among the comparison in pairs. (**E**) Hierarchical clustering of DEGs for all samples. (**F**) K-mean clustering of gene expression trends. The expression profile of each gene in each cluster is shown as a gray line, and the average expression profile of all genes in each sample is shown as blue.

RNA-seq was used to study the transcriptome changes in celery during the response to heat stress. A total of 542,097,386 raw reads were generated from all samples. After filtering, 518,610,482 high-quality clean reads were obtained, and 94.46% of filtered clean reads were mapped to the celery genome on average (Table S1). A total of 37,898 transcripts were detected, and the expression level of each gene was normalized to FPKM (fragments per kilobase of transcript sequence per million of base pairs sequenced). According to the expression level of each heat treatment, the correlation of gene expression levels between plants under any given heat treatment was calculated. The results showed that the correlation was higher for the three biological replicates within any given heat treatment than with other replicates, suggesting a differential gene expression between heat treatments (Figure 4A). Plants under QC3 showed high correlations of their gene expressions levels with those under treatment QC2 and QC4, suggesting that independently of the time spent under heat stress, a similar level of gene expressions would take place. On the contrary, plants under QC1 treatment showed the lowest correlations with heat-stressed plants.

Unsupervised PCA analysis was performed (Figure 4B), and the result showed that the four groups of celery heat stress could clearly be distinguished on the score map, indicating significant differences in the transcriptome of the four groups. One hour of heat stress showed obviously different gene expression in axis 1 (from QC1 to QC2). In addition, 6 h and 24 h of heat treatment (QC3 and QC4, respectively) mainly caused changes in the gene expression levels shown in axis 2, especially with the passage of time of heat stress (QC4). The gene expression levels shown in axis 1 almost restored to the initial state recorded without heat stress (QC1).

#### 2.4.2. Identification and Analysis of Differentially Expressed Genes

A total of 24,264 genes were differentially expressed in at least one comparison. Compared with control QC1, there were 11,376, 14,604, and 12,471 differentially expressed genes (DEGs) in QC2, QC3, and QC4 (Figure 4C). Among the six comparison pairs, the number of genes differentially expressed in QC3 vs. QC2 was the smallest, indicating that the gene expression patterns of celery after heat treatment at 1 and 6 h were similar. Five pairs (QC2 vs. QC1, QC2 vs. QC3, QC4 vs. QC3, QC4 vs. QC2, QC3 vs. QC1) were selected to analyze commonly and uniquely expressed genes in two or more comparisons (Figure 4D). A total of 1,803 DEGs were commonly expressed in the five comparison pairs. QC3 vs. QC1 showed the highest number of differentially expressed genes, indicating that the comparison of QC3 and QC1 yielded the largest number of unique genes (*n* = 1609), and the heat stress at 6 h triggered the expression of specific genes.

A hierarchical clustering heatmap was generated to view the expression patterns across the four heat stress stages (Figure 4E). It can be seen that the expression of genes could be divided into four groups, the repeatability within the three replicates in each group was good, and the differences between the groups were significant. To further classify the expression patterns of all differential genes in each sample, K-means cluster analysis was performed (Figure 4F), and four subclasses with different mean expressions were identified. Cluster 1 and Cluster 4 contained genes mainly expressed throughout heat stress. The genes of cluster 1 were highly expressed in QC2 and QC3, and the genes of cluster 4 were highly expressed in QC4. In addition, most genes showed significant changes in expression between QC2 and QC3 during hierarchical clustering and K-means clustering, suggesting that differential genes in QC2 and QC3 transcript levels could determine the heat-tolerance specificity in the celery heat-tolerance process.

#### 2.4.3. Function Annotation Analysis of DEGs

**Figure 5:**
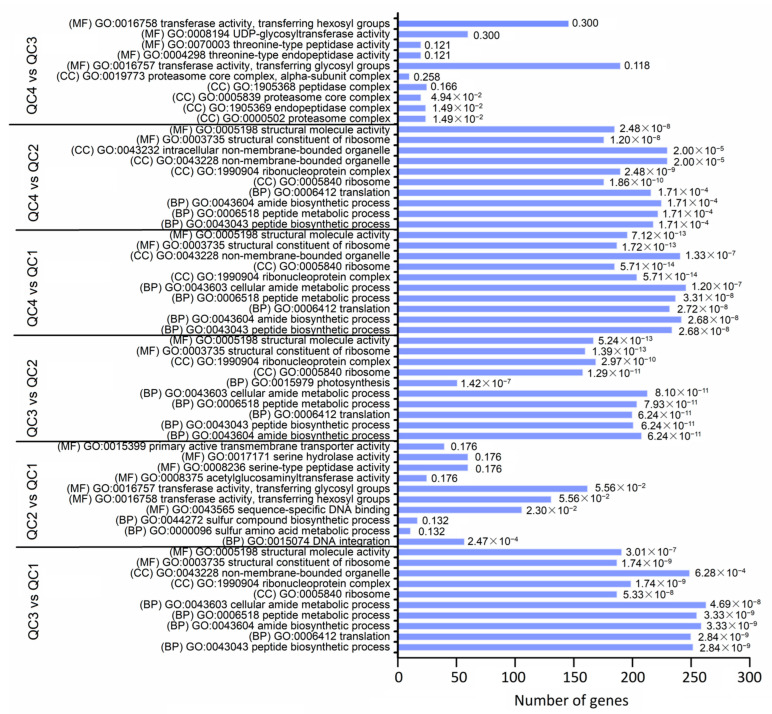
Gene Ontology (GO) enrichment analysis of DEGs among comparison pairs. The top 10 enriched terms with highly significant *p*-values (≤0.05) in each comparison are represented. The values of false discovery rate are shown in the bar graph. (BP): biological process; (MF): molecular function; (CC): cellular component.

To further analyze the functions of DEGs, gene ontology (GO) annotation was conducted on all comparison pairs. In each comparison, the top 10 enriched terms belonging to the three main GO categories of biological process (BP), cellular component (CC) and molecular function (MF) were shown (Figure 5). Four comparison pairs (QC3 vs. QC1, QC4 vs. QC1, QC3 vs. QC2, QC4 vs. QC2) were significantly enriched in BP, including “peptide biosynthesis process”, “amide biosynthesis process”, “translation” and other metabolic pathways. QC4 vs. QC3 was mainly enriched in metabolic pathways such as “proteasome complex”, “endopeptidase complex”, and “threonine-type endopeptidase activity”, indicating that these changes were not obvious after 1 h of high-temperature treatment. However, significant changes occurred in protein ribosomes at 6 h and 24 h after high-temperature treatment. According to the results of GO annotation, the biological functions and physiological processes of DEGs are comprehensively evaluated in the discussion section, and the screened DEGs associated with heat stress response may be used for further studies.

#### 2.4.4. Analysis of Differential Transcription Factors

**Figure 6:**
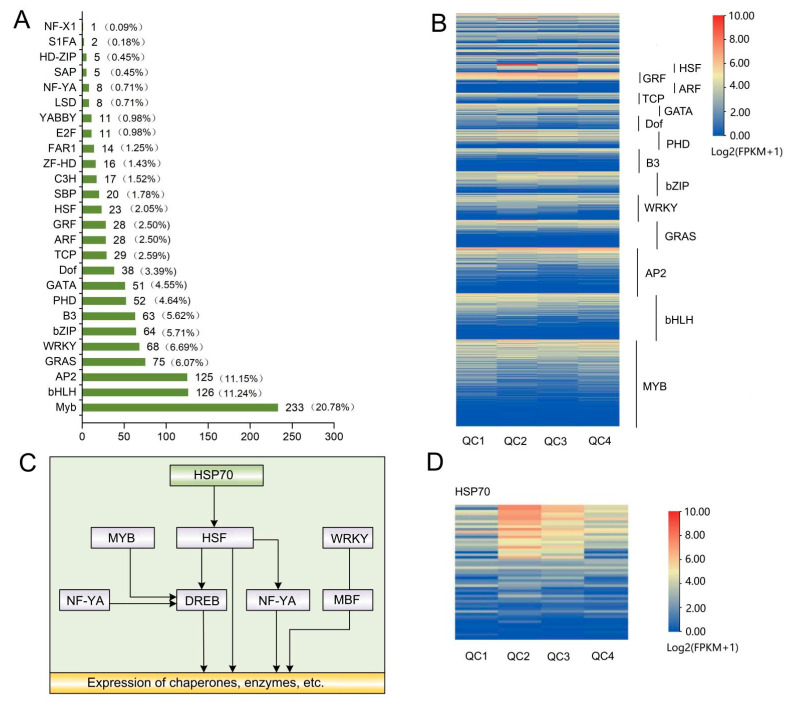
Analysis of the expression levels of transcription factors and heat shock protein (HSP) in celery under heat stress. (**A**) Differentially expressed transcription factor genes. Percentage refers to the ratio of the number of members of each transcription factor family to the total number of members of all celery transcription factors. (**B**) The specific expression of transcription factors. The color from blue to red indicates the expression level from low to high, and the expression level was calculated using log2 (FPKM + 1), and the heat map marked transcription factor families with more than 20 family members. (**C**) Correlation map of transcription families associated with heat stress. (**D**) Heat map of HSP70 protein expression, the color from blue to red indicates the expression level from low to high, and the expression level was calculated using log2 (FPKM + 1).

In this study, a total of 1121 DEGs were identified as transcription factors belonging to 26 transcription factor families, including MYB, HLH, AP2, GRAS, bZIP, WRKY (Figure 6A, Table S2). According to the gene expression of each transcription factor family (Figure 6B), the top 3 transcription factor families were MYB (*n* = 233), HLH (*n* = 126), AP2 (*n* = 125), exhibiting a high level of expression across the four stages. In addition, several families like HSF and MYB were significantly expressed in QC2 and QC3 stages.

The transcriptional families WRKY, MYB, HSF, AP2, bZIP, and NF-YA related to heat stress were studied, and an association map was established (Figure 6C). Some genes of these transcription families exhibited higher expression levels at QC2, QC3 and QC4 but lower expression levels at QC1, indicating that these transcription factors may be related to heat stress. The HSF-related heat shock protein HSP70 (Figure 6D) was significantly higher in the other three stages compared to control (QC1). Moreover, the expression under high-temperature stress for 1 h (QC2) was significantly higher than in the other three stages, suggesting that HSF and HSP70 proteins play positive regulatory roles in the heat response of celery.

### 2.5. Combined Analysis of Transcriptome and Metabolome

#### 2.5.1. Transcriptome and Metabolome Pathway Enrichment Analysis

**Figure 7:**
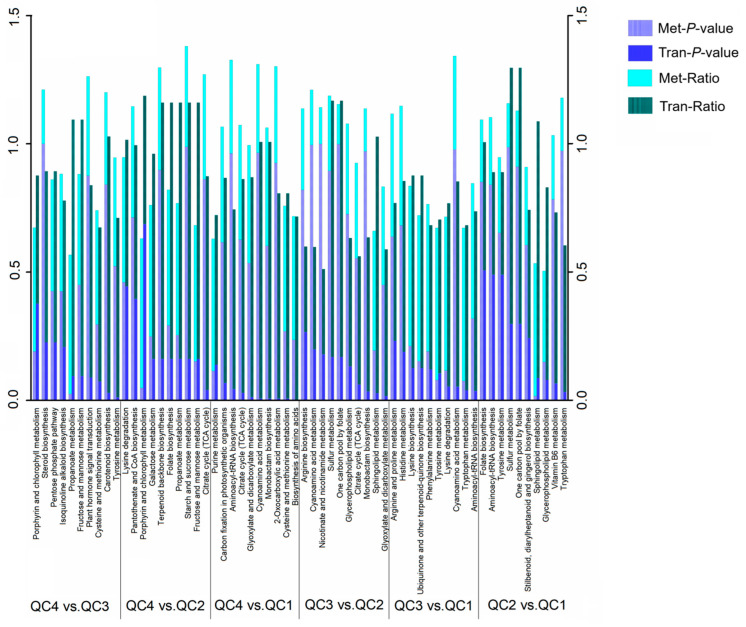
The top ten pathways associated with the transcriptome and metabolome. Ratio: differential metabolites or differential genes enriched in this pathway/number of annotated metabolites or genes in this pathway.

Significantly enriched metabolic pathways associated with DEGs and differential metabolites were found via Kyoto encyclopedia of genes and genomes (KEGG) enrichment analysis. The top ten significantly enriched metabolic pathways for each comparison pair were shown in Figure 7. From the top ten significantly enriched metabolic pathways for all six comparisons, a total of 39 metabolic pathways were identified. Highly expressed pathways in transcriptome and metabolome were “Lysine degradation”, “sphingolipid metabolism”, “phenylalanine metabolism”, “purine metabolism” and other pathways. The most significantly expressed pathways in the transcriptome were “tyrosine metabolism”, “citrate cycle (TCA cycle)”, “aminoacyl-tRNA biosynthesis”, “monobactam biosynthesis”, while the most significantly expressed ones in metabolome were “porphyrin and chlorophyll metabolism”, “sphingolipid metabolism” and other pathways. The lower the p-value value, the higher the expression level. Overall, the expression of all pathways was higher in the transcriptome than in the metabolome because the metabolome has a higher p-value than the transcriptome.

A comprehensive analysis found that the pathways affected by heat stress were mainly concentrated in “amino acid metabolism/degradation”, “citrate cycle (TCA cycle),” and “folic acid, porphyrin and chlorophyll metabolism”, which were related to amino acid metabolism, respiration and photosynthesis of celery physiological processes.

#### 2.5.2. Correlation Analysis of Transcription and Metabolism

**Figure 8:**
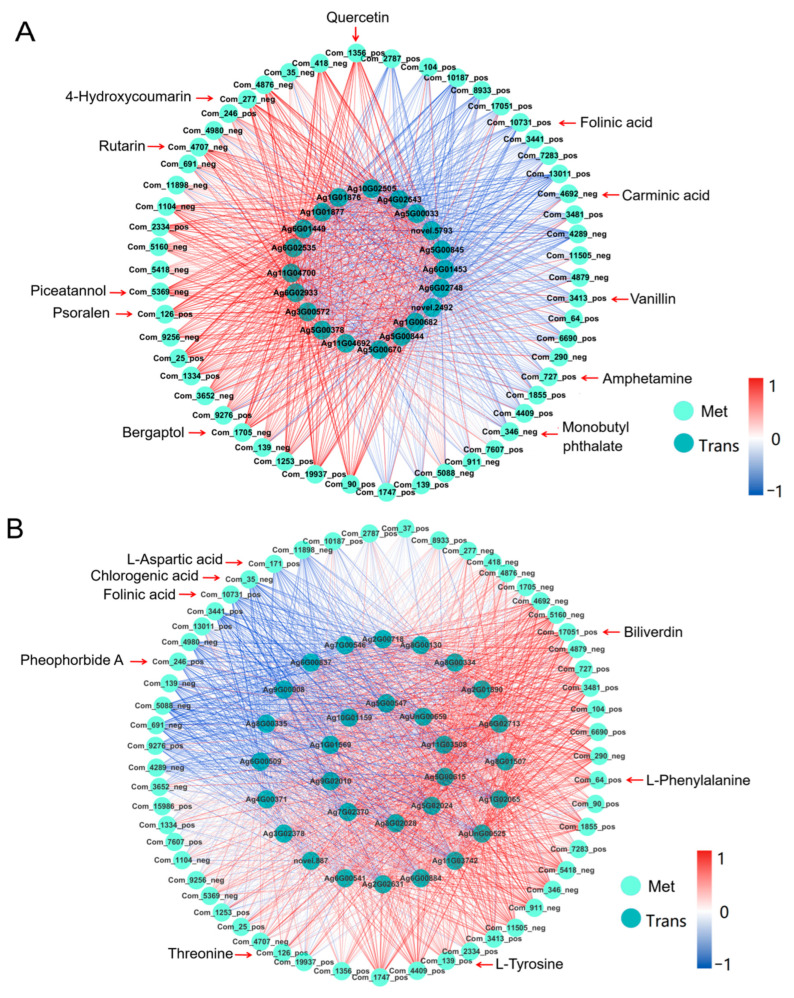
Correlation network diagram of transcriptome and metabolome. (**A**) Network diagram of correlation between HSP70 and metabolites. (**B**) Network diagram of correlation between photosynthesis genes and metabolites. Light green circles represent metabolites and the dark green represent genes. The red line represents a positive correlation, the redder the color, the higher the correlation, and the blue line represents a negative correlation, the bluer the color, the higher the correlation.

After previous analysis, it was found that celery was related to amino acid metabolism, photosynthesis, respiration, transcription family and heat shock protein HSP70 in response to heat stress, so two aspects were selected for correlation analysis. A total of 73 metabolites with different up-regulation of the top ten metabolites were selected for each comparison (Table S3). From these metabolites, metabolites related to the top ten metabolic pathways (56 in total) were selected to make the correlation diagram with HSP70 (20 with high expression were selected) and photosynthesis-related genes (30 with high expression were selected) (Figure 8). It was found that the metabolites of phenylpropanine and polyketones such as psoralen, bergaptol, 4-hydroxycoumarin, quercetin, rutarin and piceatannol were positively correlated with HSP70 and mediated by HSFs. These results indicate that the metabolites of phenylpropanoid and polyketone, HSP70 and heat shock transcription factors positively regulate response to heat stress in celery. Phenyl-type compounds such as amphetamine, monobutyl phthalate, vanillin, carminic acid and other metabolites were positively correlated with HSP70, while folinic acid and bilivedin were negatively correlated with HSP70 (Figure 8A). Amino acids (such as aspartic acid), chlorogenic acid, folinic acid, and pheophorbide A were significantly negatively correlated with photosynthesis-related genes. In contrast, amino acids such as threonine, tyrosine, phenylalanine were significantly positively correlated with photosynthesis-related genes (Figure 8B, Table S3). This finding suggests that metabolites and genes work synergistically against high temperatures.

#### 2.5.3. Transcriptome and Metabolome Analysis of Pathways Involved in Heat Stress Response

**Figure 9:**
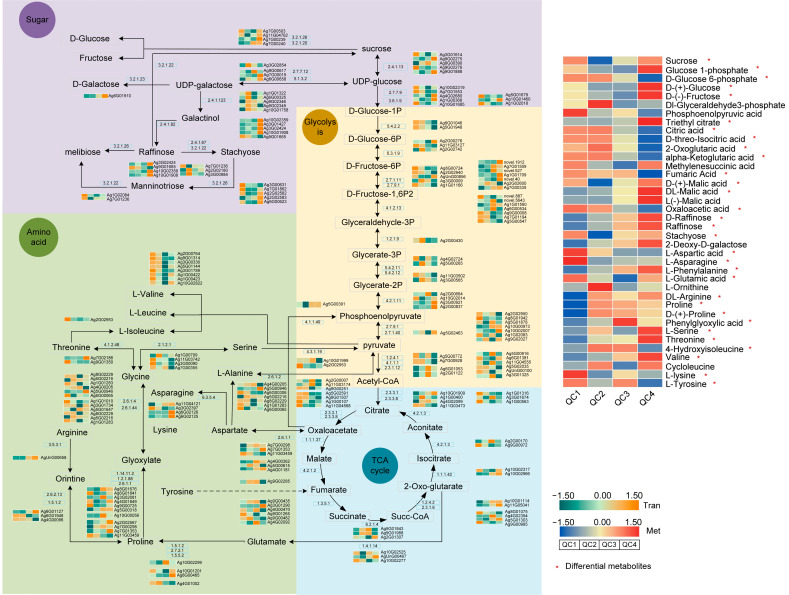
Metabolite transformation and gene expression changes of pathways (sugar, glycolysis, TCA cycle and amino acid) under heat stress. Green (down-regulated) and yellow (up-regulated) in heatmaps represent the gene expression, red (up-regulated) and blue (down-regulated) in heatmaps represent the metabolite accumulation, and the data was calculated using log2 (FPKM + 1), red asterisks represent differential metabolites.

Several pathways, including sugar metabolism, glycolysis, TCA cycle, amino acid metabolism significantly affected by heat treatment, were identified through the metabolomic and transcriptomic pathway analysis. As shown in Figure 9 and Table S4, in the glucose metabolism pathway (including 13 enzymes, 45 enzyme-encoding genes, and 11 metabolites), most of the enzyme-encoding genes were downregulated after short-term heat treatment (QC2, QC3 stage) and upregulated during QC1 and QC4 stage, including eight enzyme-encoding genes (sucrose synthase (2.4.1.13), UTP-glucose-1-phosphate uridylyltransferase (2.7.7.9), ectonucleotide pyrophosphatase (3.6.1.9), beta-fructofuranosidase (3.2.1.26), alpha-glucosidase (3.2.1.20), raffinose synthase (2.4.1.82), and stachyose synthetase (2.4.1.67)). The sucrose level of metabolites decreased by half after 1h of heat treatment and returned to normal levels after 24 h, and a three-fold increase in the expression of glucose was observed after 24 h. The sucrose level of d-glucose 1P also exhibited the same trend. Moreover, UDP-glucose--hexose-1-phosphate uridyltransferase, UDP-glucose 4-epimerase (5.1.3.2), beta-galactosidase (3.2.1.23), inositol 3-alpha-galactosyltransferase (2.4.1.123) genes were significantly upregulated after heat treatment compared with QC1. Finally, the expression of related metabolites (raffinose and stachyose) was enhanced with increased heat treatment time.

Under heat treatment, the expression of most enzyme encoding genes in glycolysis (including 14 enzymes, 52 enzyme encoding genes and 10 metabolites) and TCA cycle (including 10 enzymes, 37 enzyme encoding genes and 9 metabolites) was gradually decreased, including phosphoglucomutase (5.4.2.2), glucose-6-phosphate isomerase (5.3.1.9), diphosphate-dependent phosphofructokinase (2.7.9.1), fructose-bisphosphate aldolase (4.1.2.13), glyceraldehyde-3-phosphate dehydrogenase (1.2.1.9), 2,3-bisphosphoglycerate-independent phosphoglycerate mutase (5.4.2.12), succinate dehydrogenase (ubiquinone) iron-sulfur subunit (1.3.5.1), fumarate hydratase (4.2.1.2), citrate synthase (2.3.3.1), ATP citrate (pro-S)-lyase (2.3.3.8) and other enzymes. The related metabolites of d-glucose 6-phosphate, D-Threo-Isocitric acid, Citric acid, and alpha Ketoglutaric acid were decreased by approximately 50% at QC3 and 25% at QC4. Metabolites such as Phosphoenolpyruvic acid, 2-Oxoglutaric acid, Fumaric acid, and Oxaloacetic acid also showed a similar trend. However, 2-Oxoglutaric acid metabolites were initially increased and then decreased. Enzyme-encoding genes related to this pathway (2-oxoglutarate dehydrogenase E1 component (1.2.4.2) and succinyl-CoA synthetase alpha subunit (6.2.1.4) showed a similar trend after heat treatment.

The proline content in the amino acid metabolism pathway increased after heat treatment and was doubled at the QC2 stage, followed by a slight decrease with time. The related enzymes pyrroline-5-carboxylate reductase (1.5.1.2) and proline dehydrogenase gene exhibited the same trend after heat treatment. A three-fold increase in arginine content was observed, and gene expression of Arginase (3.5.3.1) was increased after heat treatment. Other amino acids such as serine, threonine and valine and enzymes such as Prolyl 4-hydroxylase (1.14.11.2) and aldehyde dehydrogenase (1.2.1.88) were also increased. The expression of the aspartate aminotransferase (2.6.1.1) gene increased after heat treatment, while other amino acids, such as lysine, tyrosine and aspartic acid, were downregulated. Comprehensive analysis showed that glucose, maltose, galactose and fructose in sugar metabolism were upregulated after heat treatment, glycolysis and TCA cycle intermediates were downregulated, and most amino acids were upregulated.

### 2.6. Verification of Gene Expression Levels

**Figure 10:**
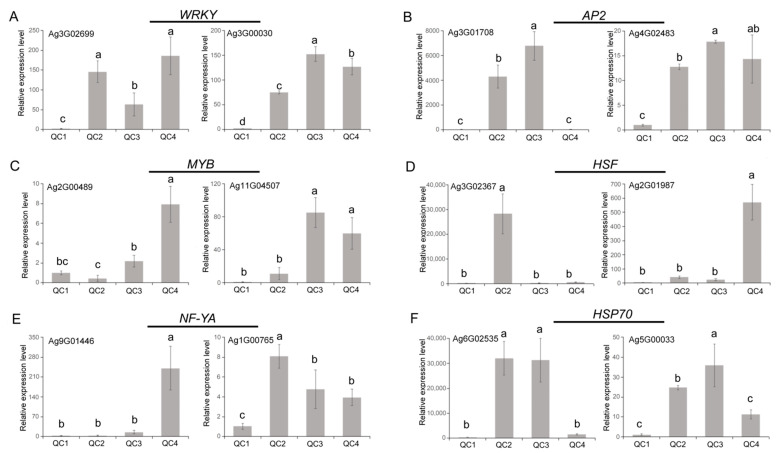
qRT-PCR analysis of celery transcription factor genes and HSPs under heat stress. The relative expression levels of WRKY (**A**), AP2 (**B**), MYB (**C**), HSF (**D**), NF-YA (**E**) and HSP (**F**). Each bar represents the mean ± SD, *T*-test at the significance level of 0.05 (*p* < 0.05), different letters (a, b, c) indicate significant difference, and the same letters indicate insignificant differences.

qRT-PCR was used to quantify mRNA levels to validate the role of genes involved in the stress response (Table S5 provides the sequences of primers used for 25 genes). In total, 12 DEGs belonging to 5 transcription factor families and the HSP70 family were selected. As shown in Figure 10, most genes were significantly upregulated after heat treatment compared with the control QC1. Genes belonging to WRKY family (*Ag3G02699*, *Ag3G00030*) and AP2 family (*Ag3G01708*, *Ag4G02483*) were significantly upregulated and maintained at high levels, *WRKY* gene expression was increased by more than 100-fold after heat treatment; the two genes of *AP2* were increased by more than 4000-fold. *MYB* was gradually upregulated after heat treatment and peaked at 6 h and 24 h, with an 8-fold increase in *Ag2G00489* and a more than 50-fold increase in *Ag11G04507*. The trend change for *NF-YA* was different after heat treatment, but both two genes were significantly upregulated after heat treatment. *HSF* gene expression was the highest after heat treatment compared with other genes. The *Ag3G02367* gene expression peaked after heat treatment for 1 h and then gradually decreased. *HSP70* expression peaked at 1 h and 6 h and then decreased, indicating that HSF/HSP mainly played an important role after heat treatment for a short time. The qRT-PCR data were consistent with the transcriptome results.

**Figure 11:**
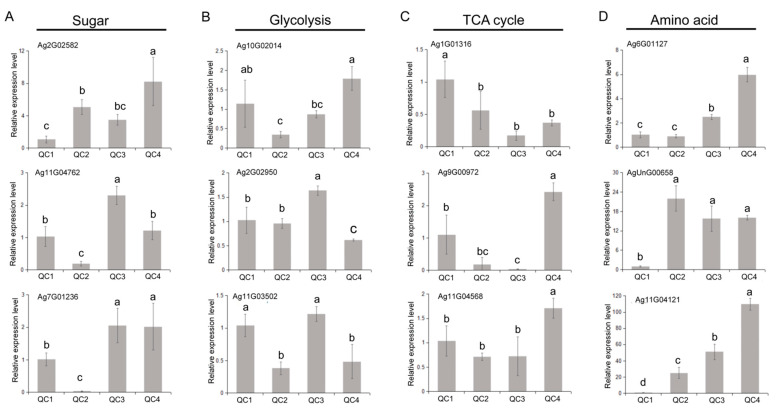
qRT-PCR analysis of genes in key pathway under heat treatment. The relative expression levels of genes in the four pathways of sugar (**A**), glycolysis (**B**), TCA cycle (**C**) and amino acid (**D**). Each bar represents the mean ± SD, *T*-test at the significance level of 0.05 (*p* < 0.05), different letters (a, b, c) indicate significant difference, and the same letters indicate insignificant differences.

Three genes related to sugar synthesis, glycolysis, TCA cycle, and amino acid metabolism were selected (*n* = 12). As shown in Figure 11 and Table S5, after heat treatment, the expression of sugar pathway genes was increased. *Ag11G04762* and *Ag7G01236* genes were significantly downregulated by more than 10-fold after 1 h of heat treatment, while a 4-fold increase in the expression of *Ag2G02582* was observed. Overall, glycolysis and TCA cycle pathway gene expression levels exhibited a downward trend. The *Ag1G01316*, *Ag10G02014*, *Ag11G04568* and *Ag9G00972* genes were downregulated more than 2-fold after heat treatment. Interestingly, the expression levels recovered at 24 h. The amino acid synthesis pathway genes were significantly upregulated after heat treatment; *Ag6G01127* and *Ag11G04121* expression increased by about 6 and 100-fold, respectively. These results were consistent with physiological, metabolome and transcriptomic findings.

## 3. Discussion

**Figure 12:**
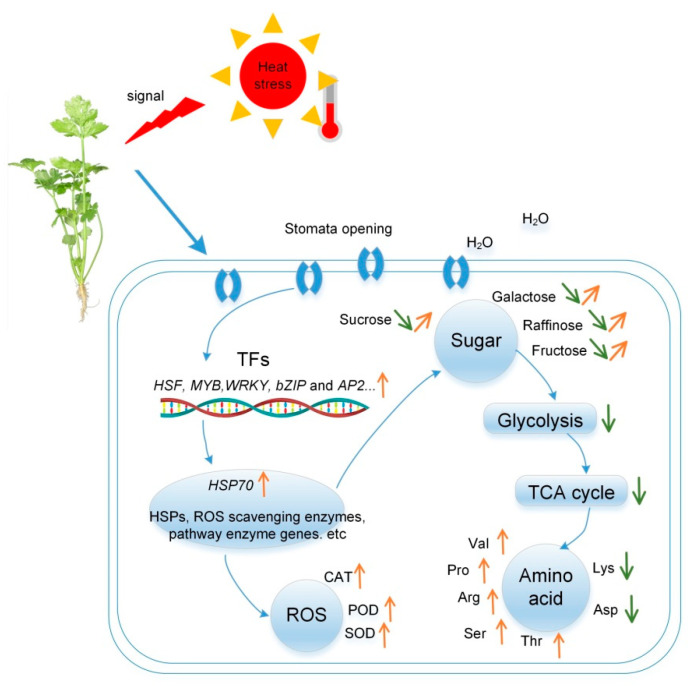
A schematic diagram summarizing the response of celery to heat stress. Changes in the main indicators of physiology, stomata, metabolome and transcription are listed. Orange arrows represent up-regulation, green arrows represent down-regulation.

High temperature has been established as an important environmental factor affecting plant growth and development, planting area and time selection. In this study, we found that heat stress could induce significant physiological, transcriptional and metabolic changes in celery leaves, increased cellular antioxidant enzyme activity, proline, glucose, fructose, etc., while decreasing chlorophyll and leaf water content (Figure 12). Plants responded to heat stress by adjusting stomata aperture. For example, high temperature could induce stomatal closure to prevent dehydration, an important protective mechanism for high-temperature tolerance [26]. In the present experiment, the stomata apertures were inclined to increase first and then decrease. After short-term heat treatment, a cooling effect was induced by an increased transpiration rate through evaporative cooling. In the case of prolonged high temperatures, plants induce stomatal closure to prevent excessive water loss. Intriguingly, it has been shown that high-temperature stress induces the production of ROS in plants [27]. High levels of ROS can have deleterious effects on plants by causing oxidative damage to macromolecules and cell membranes. Therefore, it is necessary to maintain the balance of oxidants and antioxidants such as SOD, POD, and CAT that can scavenge reactive oxygen species to maintain the survival of plants [28]. Our study found that POD and CAT activities increased significantly with time in response to high temperatures, and SOD activity was first increased and then decreased, indicating that SOD activity may significantly increase in a short time to resist high-temperature stress.

It has been established that when plants are under stress, free amino acids in cells can respond to a certain extent to adapt to the environment [29]. Amino acids can be used to synthesize proteins and provide precursors for the synthesis of various functional metabolites during plant growth and response to environmental stress [30], maintaining the osmotic balance inside and outside cells to prevent water loss [31]. Studies have shown that the proline content of poplar increased significantly under high-temperature stress [32]. In this study, proline accumulated significantly under short-term stress and decreased under long-term stress. After celery was exposed to high temperatures, the synthesis of most amino acids such as arginine and serine were upregulated, indicating that the synthesis of amino acids may be a common response to high temperature. The expression of some amino acids was also decreased; however, the types and levels of amino acids were heterogeneous in different plants under various biotic stresses [33,34].

Photosystem II is highly sensitive to high-temperature stress [35] and has been shown to affect photosynthesis and related processes in wheat in various ways [36]. High temperature can lead to a significant decrease in live leaf area and chlorophyll content [37]. Metabolome analysis in this study found that the metabolism of porphyrin and chlorophyll was related to photosynthesis and the carbon fixation of photosynthetic organisms; the expression of folinic acid biosynthesis and other pathways was decreased in the QC3 and QC4 stages. The metabolites of photosynthesis, such as Pheophorbide A, Biliverdin, etc., were low in the QC4 stage, indicating that high-temperature stress can affect photosynthesis in plants.

Gene expression in plants under heat stress is an intricate physiological and biochemical process regulated by multiple genes. In this experiment, 37,898 transcripts were obtained through transcriptome sequencing technology. After screening these genes, it was found that QC3 vs. QC1 yielded the highest number of DEGs (*n* = 14,604), and QC2 vs. QC3 the least number of DEGs (*n* = 11,108). K-means clustering analyzed the significance of changes in gene expression between QC2 and QC3. GO analysis found that DEGs were significantly enriched in biological processes, including peptide biosynthesis, amide biosynthesis, translation and other metabolic pathways. KEGG found 120 significantly enriched metabolic pathways; in QC2 vs. QC1, protein processing in the endoplasmic reticulum, fatty acid elongation and other pathways were significantly enriched. During comparison between QC3 vs. QC1, QC4 vs. QC1, QC3 vs. QC2, QC4 vs. QC2, ribosomes, photosynthesis, and chlorophyll metabolism and other pathways were significantly enriched. Combined transcriptome and metabolome analysis found that heat stress inhibited glycolysis, delayed the TCA cycle, and upregulated most amino acid synthesis pathways such as proline, arginine, and serine.

A total of 1121 differential genes were identified as transcription factors belonging to 26 transcription factor families, including the heat stress-related families HSF, WRKY, MYB, AP2, bZIP, HLH. Studies have shown that high-temperature stress transduces and activates transcription factors through various signaling pathways, inducing many HSPs and other response genes [38,39]. An increasing body of evidence suggests that high-temperature stress can promote HSP20, HSP70 and HSP90 in Arabidopsis [40], pepper [41] and poplar [15]. In this study, a total of 50 HSP70s were identified as DEGs, and HSP70s were significantly upregulated after heat stress. This phenomenon may be explained to a certain extent by the rapid response of HSP70 to heat stress; nonetheless, its expression began to decline with time. Other transcription factors, such as bZIP, DREB, MYB, WRKY, etc., may also be involved in the stress response. bZIPs are endoplasmic reticulum stress sensors that regulate abscisic acid (ABA) and stress signaling in plants, contributing to stress tolerance [6]. *WRKY40* is a positive regulator of high-temperature stress, and overexpression of *CaWRKY40* can enhance the resistance of tobacco to high-temperature stress [42]. Moreover, *WRKY6* can bind and activate the *WRKY40* promoter to regulate heat tolerance in pepper [43]. Some members of the bZIP, WRKY, HLH, MYB, and AP2 genes were initially upregulated after heat stress, suggesting that these transcription factors may play an important role in the heat shock response. Likewise, qRT-PCR results showed that these transcription factors presented similar results after heat stress. These transcription factors have been found in Arabidopsis [4], poplar [15] and other plants, but have not been found in the identification of heat-tolerant genes in celery. Considering the significant enrichment of these transcription factors and proteins under heat stress, they may be involved in the key biological processes that defend against high-temperature stress. The later verification of these transcription factors and their application is beneficial for cultivating new varieties of heat stress tolerance in celery breeding.

## 4. Materials and Methods

### 4.1. Plant Material and Treatment

The celery variety ’Jinnan Shiqin’ was used as plant material. Seeds were placed in an incubator for germination; then, germinated seedlings were transferred to growth chambers. When the plants reached the stage of about 10 leaves, 20 plants were selected for each treatment for sampling, and they were transferred to a high-temperature environment (temperature 38 °C, air humidity 80%, soil humidity about 50–60%, light intensity 15,400 lx; light/dark: 14 h/10 h) for heat stress treatment. Samples were harvested after 0 h (QC1), 1 h (QC2), 6 h (QC3) and 24 h (QC4) treatments. Celery leaf samples were collected and each treatment was sampled from different plants as a replicate. Three biological replicates were taken for the physiological and transcriptome data, and six biological replicates were taken for the metabolome. After sampling, they were quickly frozen in liquid nitrogen and stored in an ultra-low temperature freezer.

### 4.2. Measurement of Leaf Structure and Physiological Parameters

The celery leaves were added into 8 mL of 50% FAA fixating solution (formalin:glacial acetic acid:50% ethanol = 1:1:18), fixed at 4 °C for 24 h, dehydrated with ethanol, embedded in paraffin, and dyed with safranin O-fast green reagent. The leaf’s cross-sectional characteristics were observed under an optical microscope, and images were collected and analyzed by an imaging system (Nikon DS-U3, Nikon, Tokyo, Japan), stomatal features were analyzed using ImageJ software (version 1.8.0.112; Graphics software; NationalInstitutes of Health: Wayne Rasband, USA, 1997). The fresh samples were weighed and dried in an oven to a constant mass to measure their dry weights. Other samples were quickly frozen in liquid nitrogen and stored in a −80 °C refrigerator for physiological indicator determination and sequencing. The chlorophyll content was extracted by a mixture of acetone-ethanol, and electrical conductivity was measured by a conductivity meter. The MDA content was measured using the thiobarbituric acid (TBA) colorimetric method, and free proline content was measured using the ninhydrin chromogenic method. Soluble protein was assayed by coomassie bright blue G-250 staining. The SOD, CAT, and POD activities were quantified by nitrogen blue tetrazolium (NBT), the potassium permanganate titration method and guaiacol method, respectively [44,45].

### 4.3. Metabolomics Analysis

#### 4.3.1. Metabolite Extraction and Detection

Approximately 100 mg of each celery sample was transferred to a 1.5 mL centrifuge tube with 500 μL of extraction solution (methanol, water volume ratio of 4:1), followed by vortexing and incubation in an ice bath for 5 min, then centrifuging at 15,000× *g* for 20 min at 4 °C. Some supernatants were diluted to a final concentration of 53% methanol and analyzed by UHPLC-MS/MS. Samples of equal volume were taken from each experimental sample and mixed to obtain quality control samples (QC), inserted before, during and after the samples to test the repeatability of the experiment. The chromatographic and mass spectrometric conditions were as follows: a Hypesil Gold Column C18 (100 × 2.1 mm, 1.9 μm; Thermo Fisher Scientific, CA, USA) was used, the column temperature was set to 40 °C, the flow rate was 0.2 mL/min. In the positive ion mode, mobile phase A and mobile phase B components were 0.1% formic acid-aqueous and methanol, respectively. In the negative ion mode, mobile phase A and mobile phase B components were 5 mM ammonium acetate and methanol. The chromatographic gradient elution protocol consisted of: 0–12 min, 98% A, 2% B; 12–14.1 min, 100% B; 14.1–17 min, 98% A, 2% B. Mass spectrometry scan range 100–1500 *m/z*; ESI source settings: Spray Voltage: 3.2 kV; Aux Gasflow rate: 10 arb; Sheath gas flow rate: 40 arb; Capillary Temp: 320 °C; Polarity: positive, negative.

#### 4.3.2. Metabolite Data Analysis

The original file obtained by mass spectrometry was imported into Compound Discoverer (version 3.1; Thermo Fisher Scientific, TX, USA) library search software for data preprocessing. Then, the data were retrieved and compared across mzVault, mzCloud, and ChemSpider databases. Finally, the quantitative results of the screened metabolites were used for subsequent analysis.

An R package, MetaX (Wen Bo, Guangdong, China) was used for logarithmic conversion and standardized processing of the preprocessed data. After normalizing the peaks extracted from all samples, the PCA was transformed into a set of linearly uncorrelated variables through an orthogonal transformation. Differential accumulated metabolites (DAMs) were screened by three parameters, VIP (Variable Importance in the Projection), FC (Fold Change) and *p*-Value, and the thresholds were set as VIP > 1.0, FC > 1.5 or FC < 0.667 and P-value < 0.05. The annotation of the functions and classifications of the metabolites were performed against databases, including KEGG (https://www.genome.jp/kegg/pathway.html; accessed on 12 July 2021), HMDB (https://hmdb.ca/metabolites; accessed on 12 July 2021), and LIPID MAPS. Finally, the cluster heatmap, correlation and metabolic pathway analysis were conducted using the Novomagic platform.

### 4.4. Transcriptome Analysis

#### 4.4.1. RNA-Sequencing and Assembly

RNA was extracted from the four samples using the CTAB method, and the quantity and quality of extracted RNA were assessed with an Agilent 2100 bioanalyzer instrument (Agilent Technologies, CA, USA). The mRNA was separated from the total RNA by Oligo (dT) magnetic beads, and then the mRNA was randomly interrupted by divalent cations under elevated temperature. Following fragmentation, the first strand of cDNA was synthesized using random primers, followed by second-strand cDNA synthesis using DNA polymerase I. The purified double-stranded cDNA was then purified, end-repaired, and A-tailed for adapter ligation. cDNAs (370-420 bp in size) were selected by AMPure XP beads. The cDNA libraries were sequenced using the Illumina HiSeq 4000 platform with paired-end strategy (read length 150 bp) by Nuohe Gene Technology Co., Ltd. (Beijing, China).

To obtain clean sequencing data, the raw sequence reads were filtered to remove low-quality reads (reads with Qphred <=20 bases accounting for more than 50% of the entire read length), adapter sequences, and ambiguous nucleotides (N). The clean reads were separately aligned with the reference genome using HISAT2 software (version 2.0.5; Johns Hopkins University, MD, USA, 2014). New gene prediction was performed using StringTie software (version 1.3.3; Johns Hopkins University, MD, USA, 2014) [46].

#### 4.4.2. Differential Gene Analysis and Function Enrichment

The reads mapped to each gene were calculated using featureCounts (1.5.0-p3) software, and the abundance of each gene was analyzed using FPKM. DESeq2 (1.20.0) was used for differential expression analysis using |log_2_ (FoldChange)| > 0 and adjusted *p*-Value ≤ 0.05 as the screening criteria for significant DEGs [47,48]. The expression pattern of DEGs was used to represent clustering stratification and K-means clustering.

GO and KEGG functional enrichment analyses were performed on DEGs, and the related biological functions and signaling pathway analysis were done by referring to the kegg pathway map on the website (https://www.genome.jp/pathway; accessed on 15 August 2021). Combined with the plant transcription factor database PlantTFDB (http://planttfdb.cbi.pku.edu.cn; accessed on 27 August 2021), the transcription factor families of differential genes were screened and classified. The expression of these genes was calculated in log_2_ (FPKM+1), and cluster heatmaps were generated.

### 4.5. Metabolite-Gene Correlation Analysis

Using spss software (version 26; Statistical analysis software; International Business Machines Corporation: Norman H. Nie, C. Hadlai (Tex) Hull and Dale H. Bent, CA, USA, 1968.), Pearson correlation analysis was conducted between DEGs and DAMs. Then, DEGs and DAMs in the same group were mapped onto the KEGG pathway database. Cytoscape software (version 3.8.0; The Cytoscape Consortium: National Institute of General Medical Sciences: NY, USA, 2003) was used to analyze the interaction network of metabolites and genes associated with the heat stress response.

### 4.6. Validation of Gene Expression Levels

Total RNA of the samples was extracted with an RNA extraction kit (Ai Weidi Biotechnology Co., Ltd., Shenzhen, China). cDNA was synthesized by Goldenstar RT6 cDNA Synthesis Kit Ver. 2 kit (Beijing TsingKe Biotech Co., Ltd., Beijing, China). qRT-PCR was performed using CFX96TM real-time system (Bio-Rad, CA, USA) with ChamQ SYBR qPCR Master Mix Kit (Vazyme Biotech Co., Nanjing, China) reagents. The amplification procedure was 95 °C pre-denaturation for 1 min; 95 °C denaturation 10 s, annealing at 58 °C for 15 s, and the number of amplification cycles was 39 cycles. Gene expression was normalized with *AgACTIN* as an internal control and calculated with 2^−^^ΔΔCt^ [49]. The primers for qRT-PCR were designed using Primer Premier software (version 6.0; Premier Biosoft International: Palo Alto, CA), and the primer sequences are listed in Table S5.

## Data Availability

Transcriptional and metabolic data were generated by Nuohe Gene Technology Co., Ltd., and physiological and anatomic metabolic data were measured by the authors themselves.

## Supplementary Materials

The following supporting information can be downloaded at: https://www.mdpi.com/article/10.3390/ijms23063367/s1.
