# Supplementary material for: Combined Analysis of the Metabolome and Transcriptome to Explore Heat Stress Responses and Adaptation Mechanisms in Celery (Apium graveolens L.)
Source: Int J Mol Sci. 2022 Mar 20;23(6):3367. doi: 10.3390/ijms23063367 (PMC8950972; doi:10.3390/ijms23063367)
Supplement: Supplementary file 1 [file ijms-23-03367-s001.zip › Table S1.pdf]

Table S1 Transcriptome sequencing data of all samples.

sample	Raw reads	Clean reads	Clean bases	Error rate	Q20	Q30	GC content	Total map
QC1_1_1	47182330	45067148	6.76G	0.02	98.01	94.35	43.23	43214615(95.89%)
QC1_2_1	47870824	45888780	6.88G	0.02	98.06	94.42	43.53	44115560(96.14%)
QC1_3_1	43195756	41719952	6.26G	0.03	97.85	93.99	43.51	40000315(95.88%)
QC2_1_1	42878526	40922890	6.14G	0.02	97.99	94.33	43.69	38520264(94.13%)
QC2_2_1	41962100	40317306	6.05G	0.02	98.06	94.44	43.91	38215008(94.79%)
QC2_3_1	46915212	44667658	6.7G	0.02	98.04	94.49	43.66	42451987(95.04%)
QC3_1_1	45979454	43375700	6.51G	0.02	98.06	94.46	42.95	41198051(94.98%)
QC3_2_1	45603628	43876620	6.58G	0.02	98.03	94.36	42.92	41630149(94.88%)
QC3_3_1	42765048	41198832	6.18G	0.03	97.87	94	42.89	39062932(94.82%)
QC4_1_1	43893332	42303028	6.35G	0.03	97.92	94.12	42.93	38924055(92.01%)
QC4_2_1	45944036	43188718	6.48G	0.03	97.95	94.2	42.73	40183816(93.04%)
QC4_3_1	47907140	46083850	6.91G	0.03	97.96	94.21	43.12	42365287(91.93%)
