# Supplementary material for: Combined Analysis of the Metabolome and Transcriptome to Explore Heat Stress Responses and Adaptation Mechanisms in Celery (Apium graveolens L.)
Source: Int J Mol Sci. 2022 Mar 20;23(6):3367. doi: 10.3390/ijms23063367 (PMC8950972; doi:10.3390/ijms23063367)
Supplement: Supplementary file 1 [file ijms-23-03367-s001.zip › Table S5.pdf]

Table S5 Sequences of primers used in qRT-PCR.

ID	Forward primer (5'→3')	Reverse primer (5'→3')
Ag3G02699	ACTCCTGCGTCCAGCACCAT	GCTCTCTGCTACCACCATCGTC
Ag3G00030	GCACGAGACAGAGCCAGTCAAT	GCAGAAGGAGCGGTTCCATGTT
Ag3G01708	TCCAAGCCCAGATCCAACTCCA	ACCAAAGTCGGGTTCGGTTCTT
Ag4G02483	CATAACCCGACCCGGCTCTTTG	AGCTTCGTGGGCTTTGGTGG
Ag2G00489	TCCACTGTTGAGGCTCCACTGA	AGGAGGTAGGTTGCCACCATCA
Ag11G04507	TGGACCGAAGAGGAGCACAGAT	GGTGGAGGGACTGGTTGGGTAT
Ag2G01987	CCGCTTGATCTGATGCCGTTGA	TCCAGCCCACCGCTTATCTTGA
Ag3G02367	GCGAGAACGAGCGGTTGAGAA	CCTGCTGCTGTTGCGAACTACT
Ag9G01446	CCATCTCGGCTGCTTCAACTCA	GAGGTGGTCCCTTCTTGGTTCC
Ag1G00765	AACCCACCTCAGGCTCCACAAA	GCACGGTACTGCTTGGCATTCA
Ag6G02535	GGCGTCTGGCAACACGATAGAG	AGCAGCGTCACCAATCAACCTT
Ag5G00033	ACCCGACCCGAACCAACTCAA	TTCTCCGCCGCCACTCGTAA
Ag2G02582	GCTCAGCGGCACAGTTAGACAT	AGAGAGTGGGTCGTCAGCAAGA
Ag10G02319	TCCAGACTCCGACCGATGAAGT	CGTGCCCAGTCCTCCATTAAGC
Ag7G01236	TGGCATCGGCTTAACACCTCAG	CGTTGAGCGTCTAGGCACCAT
Ag10G02014	GCTGATTTGGCTGTCGGTTTGT	GGCATACGGAAGTTGGCTCCTG
Ag2G02950	TTGTGCCGAGGCAGAGAAGGTT	CCGTACCGCTGACGAAGCAATG
Ag11G03502	GGGCTCATGGAACTGCTGTTGG	ACCTCGCTCACTGGCACCTT
Ag1G01316	TGCTCCACTGGTTGCGACTCT	ACTCTTCCGAAGGGCAATGGGA
Ag9G00972	TGCCGCAACGAGGTACAAGGA	CCTGGCTTCAACTCGCTGACTT
Ag11G04568	TTGAAGGCGTTGCGATGGAACT	CCATTCGGCATCCTGGAACACT
Ag6G01127	TGCTGGCTTGTGACTGGGAAGA	CCATGCTCTCCTGGCTGTATGC
AgUnG00658	GCTTGTTCGGGCTTTAGGAGGT	CGCAGCGGATCTTCTTCCATCA
Ag11G04121	TGTGGTCACCGTCAATGGAGAG	CCAATGGCATCACGAGCAACAA
Actin	AGAAGTCCTGTTCCAGCCGTCTT	CGAACCACCACTGAGCACTATGTT
